# Disrupted upregulation of salience network connectivity during acute stress in siblings of schizophrenia patients

**DOI:** 10.1017/S0033291719004033

**Published:** 2021-04

**Authors:** Judith M. C. van Leeuwen, Christiaan H. Vinkers, Matthijs Vink, René S. Kahn, Marian Joëls, Erno J. Hermans

**Affiliations:** 1 Department of Psychiatry, University Medical Center Utrecht, Utrecht, The Netherlands; 2 Donders Institute for Brain, Cognition, and Behaviour, Radboud University Medical Center, Nijmegen, The Netherlands; 3 Department of Psychiatry/GGZ InGeest, Amsterdam UMC (location VUmc), Amsterdam, the Netherlands; 4 Department of Anatomy and Neurosciences, Amsterdam UMC (location VUmc), Amsterdam, the Netherlands; 5 Utrecht University, Experimental Psychology, Utrecht, The Netherlands; 6 Department of Psychiatry, Icahn School of Medicine at Mount Sinai, New York, New York, USA; 7 Department of Translational Neuroscience, University Medical Center Utrecht, Utrecht, The Netherlands; 8 University of Groningen, University Medical Center Groningen, Groningen, The Netherlands

**Keywords:** Cortisol, large-scale functional brain networks, neuroimaging, resilience, schizophrenia, stress

## Abstract

**Background:**

An adaptive neural stress response is essential to adequately cope with a changing environment. It was previously argued that sympathetic/noradrenergic activity during acute stress increases salience network (SN) connectivity and reduces executive control network (ECN) connectivity in healthy controls, with opposing effects in the late aftermath of stress. Altered temporal dynamics of these networks in response to stress are thought to play a role in the development of psychopathology in vulnerable individuals.

**Methods:**

We exposed male healthy controls (*n* = 40, mean age = 33.9) and unaffected siblings of schizophrenia patients (*n* = 39, mean age = 33.2) to the stress or control condition of the trier social stress test and subsequently investigated resting state functional connectivity of the SN and ECN directly after and 1.5 h after stress.

**Results:**

Acute stress resulted in increased functional connectivity within the SN in healthy controls, but not in siblings (group × stress interaction *p*_fwe_ < 0.05). In the late aftermath of stress, stress reduced functional connectivity within the SN in both groups. Moreover, we found increased functional connectivity between the ECN and the cerebellum in the aftermath of stress in both healthy controls and siblings of schizophrenia patients.

**Conclusions:**

The results show profound differences between siblings of schizophrenia patients and controls during acute stress. Siblings lacked the upregulation of neural resources necessary to quickly and adequately cope with a stressor. This points to a reduced dynamic range in the sympathetic response, and may constitute a vulnerability factor for the development of psychopathology in this at-risk group.

## Introduction

While a substantial body of research has established an important role for stress in the development of many psychiatric disorders (Butjosa et al., 2016; Koenders et al., 2014; Lex, Bäzner, & Meyer, 2017; McCraw & Parker, 2017; Shapero et al., 2017), not all people who experience stressful life events develop a form of psychopathology (Kalisch et al., 2017). The neural mechanisms underlying these interindividual differences are not well understood, but have previously been suggested to arise from impaired temporal dynamics of large-scale brain networks in response to stress in vulnerable individuals (Homberg, Kozicz, & Fernández, 2017).

Stress initiates a cascade of temporally distinct neurochemical and physiological changes, which allow individuals to deal with an ongoing stressor and return to homeostasis thereafter. In this context, Hermans and colleagues proposed an integrative model of the acute and delayed effects of stress on large-scale neural networks in the healthy brain (Hermans, Henckens, Joëls, & Fernández, 2014). First, the activation of the sympathetic nervous system and the release of catecholamines such as (nor)epinephrine and dopamine shift the brain to a hyperalerted state. This is accompanied by increased activity within, and connectivity between, nodes of the salience network (SN), including the anterior insula, dorsal anterior cingulate cortex (dACC), and amygdala (Hermans et al., 2014, 2011; van Marle, Hermans, Qin, & Fernández, 2010). Supraoptimal levels of catecholamines simultaneously reduce functioning of the executive control network (ECN), impairing complex cognitive abilities during a stressful event (Arnsten, 2009; Qin, Hermans, van Marle, Luo, & Fernández, 2009; Shields, Sazma, & Yonelinas, 2016), and promoting habitual behavior as a result of attenuated top-down control over limbic areas (Schwabe, Tegenthoff, Höffken, & Wolf, 2010).

While these rapid changes are considered to form an adaptive response to acute stressors, their adequate termination is essential for successful recovery from stress (de Kloet, Meijer, de Nicola, de Rijk, & Joëls, 2018). Levels of cortisol, the end-product of the hypothalamus-pituitary-adrenal axis, peak around 30 min after stress (Dickerson & Kemeny, 2004). While cortisol, through nongenomic pathways, amplifies catecholaminergic effects in the early phase of the stress response (de Kloet, Karst, & Joëls, 2008), slower genomic effects of cortisol are thought to aid the return to homeostasis by reversing the hyperalerted state and enhancing cognitive processes such as reappraisal of the situation after stress has subsided (Henckens, van Wingen, Joels, & Fernandez, 2010; Hermans et al., 2014; Joëls, Sarabdjitsingh, & Karst, 2012). The administration of hydrocortisone (a synthetic form of cortisol) slowly reduces activity and connectivity of the amygdala (Henckens et al., 2010; Henckens, van Wingen, Joëls, & Fernández, 2012a), improves emotional states (Reuter, 2002), and enhances dlPFC functioning and working memory performance (Henckens et al., 2012), supporting a role for the late effects of cortisol on SN and ECN functioning.

Even in the absence of stress, SN and ECN functional connectivity was found to be affected in severe psychiatric disorders including schizophrenia (Negrón-Oyarzo, Aboitiz, & Fuentealba, 2016; Wang et al., 2016). Moreover, increased SN functional connectivity is associated with subclinical symptoms in individuals at clinical high risk for psychosis (Pelletier-Baldelli, Bernard, & Mittal, 2015) and increased insula activity is associated with auditory and verbal hallucinations in schizophrenia (Sommer et al., 2008). Given the time-dependent effects of stress on the SN and ECN, altered functional connectivity of these two networks in psychiatric disease could be the result of a maladaptive response to stress in vulnerable individuals (Homberg et al., 2017). However, no studies to date have investigated the temporal dynamics of these two large-scale brain networks in response to stress in an at-risk group.

Here, we investigated the acute and delayed effects of an acute stressor, the trier social stress test (TSST), on functional connectivity of the SN and ECN during task-free periods in healthy controls and unaffected siblings of schizophrenia patients. Siblings have a higher chance to develop various psychiatric disorders, including schizophrenia, bipolar disorder, and major depressive disorder (Cheng et al., 2017), and show increased stress sensitivity in daily life (Myin-Germeys, Marcelis, Krabbendam, Delespaul, & van Os, 2005). We hypothesized that stress would immediately result in increased SN functional connectivity and decreased ECN functional connectivity in healthy controls, whereas we expect to find the opposite in the aftermath of stress. Moreover, we examined whether there are differences between siblings and controls, expecting a potential increase in SN functional connectivity during stress and/or attenuated recovery of this network in the aftermath of stress. Finally, we expected an increase in cortisol levels and adrenergic activity after stress and investigated potential differences between the groups.

## Methods and materials

### Participants

We recruited 40 healthy controls and 40 healthy siblings of schizophrenia patients from the Genetic Risk & Outcome of Psychosis (GROUP) study (Korver, Quee, Boos, Simons, & de Haan, 2012) and via advertisements. Because of the influence of gender and the menstrual cycle on stress-induced cortisol levels (Kirschbaum, Kudielka, Gaab, Schommer, & Hellhammer, 1999) we only included male participants. Participants in the control group were matched to siblings based on demographical data and both groups were subsequently randomly assigned to the validated stress or no-stress condition of the TSST (see below for detailed description). None of the participants suffered from a psychiatric disorder [as assessed with a semi-structured interview by a trained researcher (the Mini-International Neuropsychiatric Interview (Sheehan et al., 1998))], used any synthetic corticosteroids, carried ferromagnetic objects in their body, or suffered from claustrophobia. Furthermore, controls did not have first-degree relatives with a psychiatric disorder. The current use of psychoactive substances (amphetamines, cocaine, opiates, methadone, benzodiazepines, and cannabinoids) was determined with a urine multi-drug screening device (Multi-line) and self-report questionnaire. None of the participants reported the use of drugs in the past 3 days. Two participants (1 control and 1 sibling) tested positive for cannabis. Exclusion of these participants did not influence any of the results. One participant was excluded due to technical problems with the magnetic resonance imaging (MRI)-scanner (sibling-stress group). This resulted in four experimental groups: control-no-stress (*n* = 20), control-stress (*n* = 20), sibling-no-stress (*n* = 20), and sibling-stress (*n* = 19). Differences in demographics between the four groups were analyzed using one-way analyses of variance (ANOVAs) or χ^2^ tests using SPSS 23.0 (Statistical Package for the Social Sciences, Chicago, IL).

Prior to the experiment, all participants gave written informed consent. The authors assert that all procedures contributing to this work comply with the ethical standards of the relevant national and institutional committees on human experimentation and with the Helsinki Declaration of 1975, as revised in 2008.

### General procedures and stress induction

We told participants that the study investigated the effects of ‘cognitive load’ on the brain. We provided all information regarding the study purpose during debriefing. Participants were instructed to refrain from heavy exercise (2 h prior to participation) and caffeine intake (4 h prior to participation). The experimental scan session started with an anatomical and resting state scan (RS0) to acclimatize all participants to the scanning environment. The first scan session was followed by the stress or control condition of the TSST. The first post-stress resting state (RS1) was acquired 20 min following TSST onset, followed by an emotion processing task [viewing and rating pictures from the international affective picture system (van Leeuwen et al., 2018)] and a reward processing task (van Leeuwen et al., 2019). The second post-stress resting state scan (RS2) was acquired 90 min following TSST onset. The TSST was carried out as previously published (Kirschbaum, Pirke, & Hellhammer, 1993), between 4:30 and 8:30 PM to minimize variation in diurnal cortisol secretion. In short, participants received instructions 5 min prior to the stress or control condition, which was carried out outside the scanner in a separate room. The stress condition consisted of a 5 min job interview, followed by a 3 min mental arithmetic task in front of a committee (one woman and one man). The control condition consisted of a free speech (5 min) followed by a simple arithmetic task (3 min) (Het, Rohleder, Schoofs, Kirschbaum, & Wolf, 2009). The experimenter was in the same room but did not evaluate the participant, nor was there a committee present.

### Cortisol and subjective stress

We obtained seven saliva samples throughout the experiment using salivettes (Sarstedt, Nümbrecht, Germany) for the quantification of cortisol and alpha-amylase [an indirect marker of adrenergic activity (van Stegeren, Rohleder, Everaerd, & Wolf, 2006)] at the following time points: −10, +5, +20, +30, +65, +90, and +120 min relative to TSST onset. Samples were temporarily stored at 4 °C and subsequently stored at −20 °C. Cortisol and alpha-amylase levels were analyzed as described previously (Vinkers et al., 2013). In short, cortisol was measured without extraction using an in-house competitive radio-immunoassay, and alpha-amylase was measured using a Beckman-Coulter AU5811 chemistry analyzer. Three out of 497 cortisol samples were not collected (all non-peak values) and these missing values were calculated based on the mean group decline. The area under the curve (AUCi) for cortisol was calculated as previously described (Pruessner, Kirschbaum, Meinlschmid, & Hellhammer, 2003). The effects of time, stress, group, and their interaction on cortisol and alpha-amylase levels were analyzed using repeated measures ANOVAs using SPSS 23.0.

### Structural and functional MRI acquisition

All imaging was performed on a Philips 3.0-T whole-body MRI scanner (Philips Medical Systems). First, a whole-brain three-dimensional T1 weighted structural image was acquired with the following scan parameters: voxel size 1 mm isotropic; repetition time (TR) = 10 ms; echo time (TE) = 4.6 ms; FOV = 240 × 240 × 160 mm; flip angle = 8°. Functional images were obtained using a two-dimensional echo planar imaging-sensitivity encoding sequence with the following parameters: voxel size 3 mm, slice thickness = 3 mm, TR = 2000 ms; TE = 35 ms; 35 slices; gap = 0.43 mm; flip angle = 70°. Two hundred and two functional scans were acquired during the resting state scan (acquisition time: 7 min). A small white cross on a black background was shown for the entire scan duration. Participants were instructed to lay still, keep their eyes open and stay awake.

### Preprocessing

We performed preprocessing using the tools from the Oxford Centre for Functional MRI of the Brain (FMRIB) Software Library [FSL; http://www.fmrib.ox.ac.uk/fsl; RRID:SCR_002823; (Smith et al., 2004)]. First, correction for head movement was done using fMRI Expert Analysis Tool (FEAT) and Independent Component Analysis-based Automatic Removal of Motion Artifacts [ICA-AROMA; (Pruim et al., 2015)] after which a high pass filter of 128 s was applied. Then, the functional images were coregistered to the anatomical image and normalized in SPM12 (http://www.fil.ion.ucl.ac.uk/spm) using unified segmentation (Ashburner & Friston, 2005). All images were spatially smoothed using an 8-mm FWHM Gaussian kernel.

Given (1) the between-subject design, allowing a direct comparisons between functional connectivity during stress and no-stress conditions and (2) the portion of participants that had never been in an MRI-scanner before the experiment [which is stressful in itself (Muehlhan, Lueken, Wittchen, & Kirschbaum, 2011)], we focused on the two post-stress resting state scans in this paper and disregarded RS0. Indeed, cortisol levels were higher directly after RS0 for scanner-naïve participants (online Supplementary Fig. S1B), and scanner-related subjective stress was highest before the first MRI-scan (online Supplementary Fig. S1A), suggesting that in general the first functional scan session of an experiment should not be used as baseline resting-state scan but rather to acclimatize participants to the scanner environment.

### Functional connectivity

To analyze network connectivity on the subject-level, we extracted and averaged the time series from all voxels within pre-defined core nodes of the salience and ECNs (network signal) (Shirer, Ryali, Rykhlevskaia, Menon, & Greicius, 2012). The SN seeds consisted of the dACC and bilateral anterior insula. The ECN consisted of the bilateral dorsolateral prefrontal and bilateral posterior parietal cortices. For all seeds, we selected only voxels within gray matter. Timeseries extraction was performed on unsmoothed data. The averaged timeseries of each of the two networks and each of the two sessions was fed into a separate first-level ANOVA, resulting in four connectivity maps per participant (two sessions and two networks). We opted not to include additional regressors since nuisance signals (e.g. motion-related signal fluctuations) were already removed from the data using the ICA-AROMA preprocessing step explained above.

To investigate the acute and delayed effects of stress on SN and ECN connectivity in both groups, we applied two second level full-factorial ANOVAs with stress (stress/no-stress) and group (control/sibling) as between-subject factors: one with the connectivity maps from RS1 and one from RS2. Statistical parametric maps were thresholded at a cluster-level whole-brain family-wise error (FWE) corrected threshold of *p* < 0.05 (cluster-forming threshold at the voxel-level: *p* < 0.001, uncorrected). For hypothesis-driven investigations of network changes under acute stress in regions of interest (ROIs) for which we had a-priori hypotheses, we applied small-volume corrections with search regions. We identified the left and right insula and the dACC as ROIs for the SN analyses, and the left and right middle frontal gyrus and the left and right parietal cortex (a combination of the inferior parietal and the angular gyrus) for the ECN analyses. All ROIs were based on the automatic anatomical labeling atlas (AAL, Tzourio-Mazoyer et al., 2002).

## Results

### Demographics

The four groups did not differ in age, BMI, education, and self-reported childhood trauma (Table 1).

**Table 1.** Group characteristics

	Con-no-stress *N* = 20	Con-stress *N* = 20	Sib-no-stress *N* = 20	Sib-stress *N* = 19	*p*^a^
Age	33.05 (1.91)	34.80 (2.03)	33.85 (2.42)	32.53 (1.70)	0.87
Education	7.55 (0.34)	7.10 (0.42)	7.00 (0.35)	7.37 (0.35)	0.71
BMI	24.35 (0.64)	24.19 (0.46)	24.00 (0.68)	24.90 (0.89)	0.81
Handedness (right/left/both)	18/2/0	19/1/0	14/4/2	17/2/0	0.84
Childhood trauma (CTQ total score)	34.50 (2.00)	35.70 (3.00)	36.15 (2.19)	34.0 (1.40)	0.89

Con, control participant; Sib, sibling of schizophrenia patient.

Mean values (standard error of the mean) are denoted for age, education, body mass index (BMI), and childhood trauma. Handedness is reported in total right/left/both hand preference.

a Comparisons were made between the four groups.

### Cortisol, alpha-amylase, and subjective stress

**Fig. 1.**
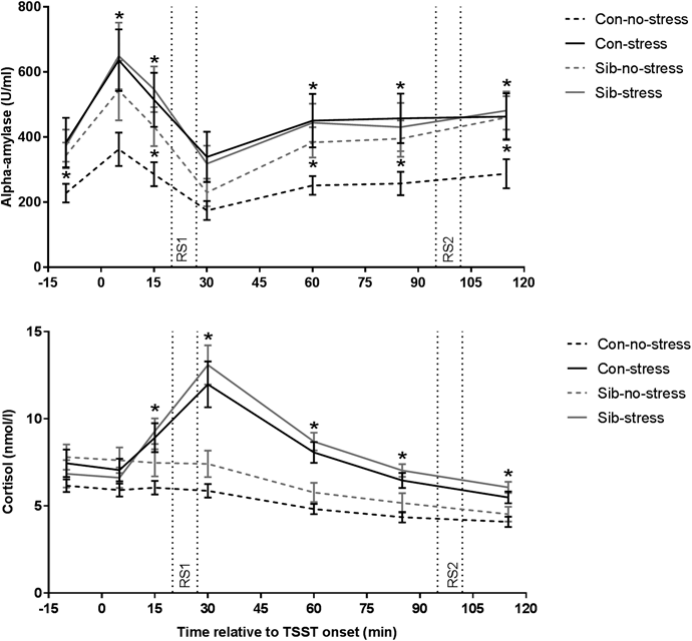
Salivary alpha-amylase and cortisol responses to stress. Con = healthy control, Sib = sibling of schizophrenia patient. * *p* < 0.05. Error bars represent standard error of the mean (s.e.m.).

First, we validated successful stress induction by the TSST. Indeed, the TSST affected salivary cortisol levels over time (time × stress interaction *F* = 12.401, *p* = 1.833 × 10^−9^, *η*_p_^2^ = 0.515) and the area under the curve was larger for stress than control sessions (*F* = 21.621, *p* = 1.4 × 10^−5^, *η*_p_^2^ = 0.224). Time-dependent changes appeared to be different between controls and siblings (time × group × stress interaction *F* = 2.310, *p* = 0.043, *η*_p_^2^ = 0.165) but follow-up analyses did not reveal significant differences on individual samples (stress × group interaction on all assessments *p* > 0.05). Moreover, the AUCi for stress *v.* no-stress sessions, a robust measurement of stress reactivity, did not differ between controls and siblings (stress × group interaction *F* = 2.240, *p* = 0.139, *η*_p_^2^ = 0.029), indicating that indeed there was no systematic difference between healthy controls and siblings of schizophrenia patients in stress-induced cortisol concentrations (Fig. 1).

We then investigated the effect of the TSST on alpha-amylase levels, an indirect marker for adrenergic activity (van Stegeren et al., 2006). Overall, alpha-amylase concentrations were higher in the stress compared to the no-stress condition (main effect of stress *F* = 5.757, *p* = 0.019, *η*_p_^2^ = 0.074, main effect of group or group × stress interaction *p* > 0.05). We found a trend towards a similar effect of the TSST on alpha-amylase AUCi (*F* = 3.736, *p* = 0.057, *η*_p_^2^ = 0.047, main effect of stress and group × stress interaction *p* > 0.05). Focusing on the no-stress condition only, we found that siblings of schizophrenia patients had higher alpha-amylase levels throughout almost the entire experiment (sibling *v.* control in the no-stress condition *p* < 0.05 at *t* = −10, 15, 60, 85, and 115 min relative to TSST onset), while there was no difference between siblings and controls in the stress condition, or between siblings in the no-stress condition and the stress condition (all comparisons *p* > 0.1). These results demonstrate increased alpha-amylase concentrations in response to the TSST across both participant groups and higher alpha-amylase levels in the absence of stress in siblings (Fig. 1).

Subjective stress increased in response to the TSST (time × stress interaction *F* = 7.911, *p* = 0.00078, *η*_p_^2^ = 0.178). These ratings did not differ between healthy controls and siblings (time × group × stress interaction *F* = 0.841, *p* = 0.435, *η*_p_^2^ = 0.023).

### Salience network functional connectivity

**Fig. 2.**
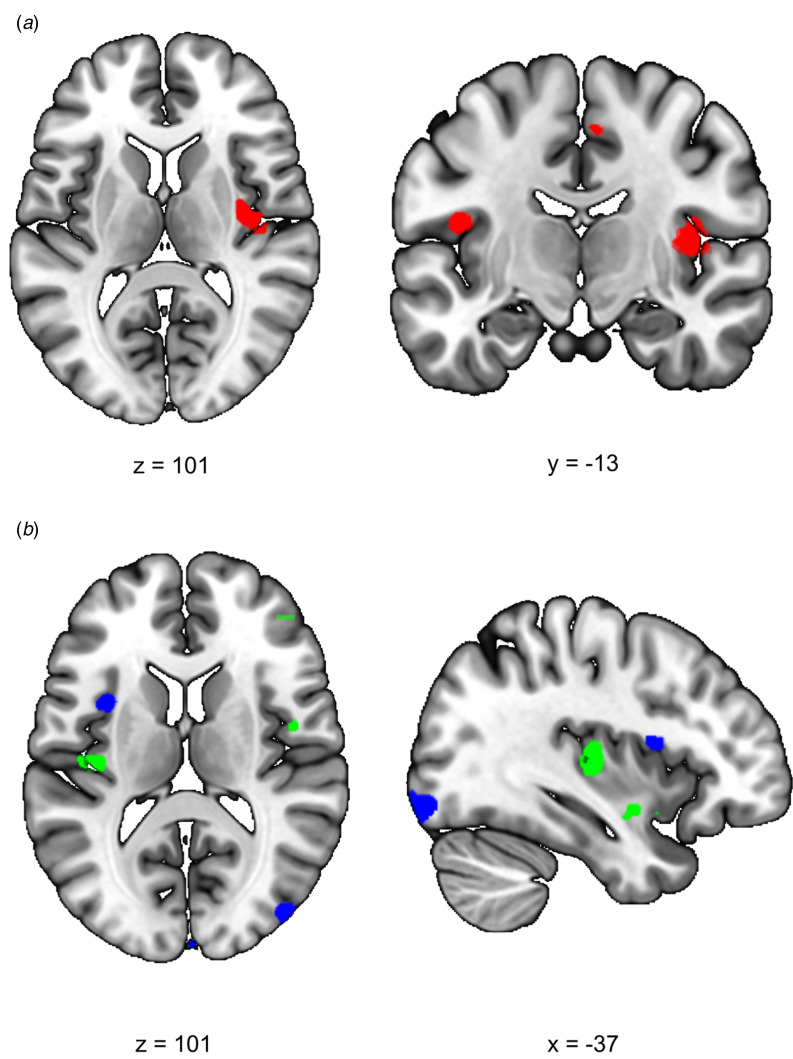
Effects of stress on SN functional connectivity in siblings of schizophrenia patients and controls. (*a*) Group (control/sibling) × stress (stress/no-stress) interaction in SN connectivity during acute stress. Controls and siblings differed in SN connectivity directly after stress (group × stress interaction (*p*_svc_ < 0.05)) in the right insula. Follow up comparisons revealed that this was driven by a stress-induced increase in healthy controls but not in siblings. (*b*) Reduced SN connectivity in the aftermath of stress (blue) and a group (control/sibling) × stress (stress/no-stress) interaction (green) in the aftermath of stress. Functional connectivity between the SN and the right anterior insula decreased in the aftermath of stress in both healthy controls and siblings of schizophrenia patients (blue, no-stress > stress *p*_svc_ = 0.048). Additionally, there was a decrease in functional connectivity between the SN and the posterior insula in the aftermath of stress in siblings (green, group × stress interaction *p*_svc_ = 0.032 and *p*_svc_ = 0.057). *T*-Maps are thresholded at *p* < 0.001 uncorrected and overlaid onto a normalized anatomical scan for visualization purposes. For a color version, see this figure online. See Table 2 for cluster level inferences.

We first investigated the acute response to stress (RS1), expecting increased SN functional connectivity in healthy controls. We found a significant difference between siblings and controls in stress-induced functional connectivity between the right insula and the rest of the SN (group × stress interaction *T* = 4.10, cluster size = 1120 mm^3^, *p*_svc_ = 0.008) which survived whole brain correction as well (cluster size = 2808 mm^3^, *p*_whole−brain_ = 0.046) (Fig. 2*a*). *Post-hoc* comparisons between the groups indeed revealed a significant increase in functional connectivity between the insula and the rest of the SN during stress relative to no-stress in controls (con-stress > con-no-stress *T* = 3.88, cluster size = 896 mm^3^, *p*_svc_ = 0.012) but not in siblings (sib-stress > sib-no-stress; no suprathreshold clusters). Moreover, controls in the stress condition had higher connectivity levels than siblings in the stress condition at the trend level [con-stress > sib-stress (*T* = 3.43, cluster size = 24 mm^3^, *p*_svc_ = 0.093; *T* = 3.30, cluster size = 32 mm^3^, *p*_svc_ = 0.089)], supporting increased SN functional connectivity in stressed controls relative to stressed siblings, rather than a difference between the two groups during no-stress conditions. These findings indicate an increase in SN functional connectivity during stress in healthy controls, and a lack of SN reactivity in siblings.

We expected to find decreased SN functional connectivity in the late aftermath of stress (RS2, 90 min after TSST onset) in healthy controls, which would be affected in siblings. We found a group × stress interaction in the left insula, indicating a difference between the groups in functional connectivity between this area and the rest of the SN (*T* = 3.82, cluster size = 400 mm^3^, *p*_svc_ = 0.032; *T* = 3.54, cluster size = 168 mm^3^, *p*_svc_ = 0.057) (Fig. 2*b*). Follow up comparisons revealed a decrease in this area in siblings in the stress condition compared to the no-stress condition (sib-no-stress > sib-stress *T* = 3.85, cluster size 288 mm^3^, *p*_svc_ = 0.041; *T* = 3.45, cluster size = 368, *p*_svc_ = 0.034) and no difference between controls in the stress and no-stress condition (no suprathreshold clusters). Additionally, we found a main effect of stress on functional connectivity with the left anterior insula, with decreased levels in the stress groups (no-stress > stress *T* = 3.97, cluster size = 232 mm^3^, *p*_svc_ = 0.048), which did not differ between siblings and controls (no suprathreshold clusters). These results support a general SN downregulation in the aftermath of stress in both groups, and an additional disconnect with the posterior insula in siblings only.

### Executive control network functional connectivity

We first investigated ECN functional connectivity during the acute phase of stress (RS1), expecting decreased connectivity in healthy controls in the stress condition. However, we found no significant differences between the groups on ECN functional connectivity during this phase (stress > no-stress, no-stress > stress, group × stress interaction all *p* > 0.05).

**Fig. 3.**
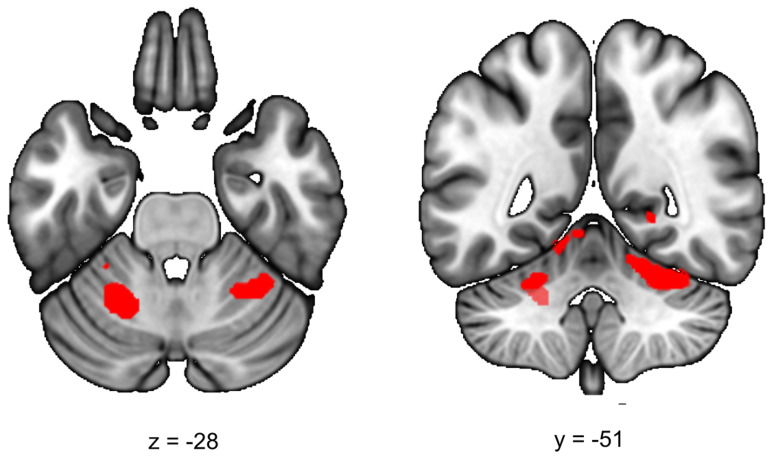
Executive control functional connectivity in the aftermath of stress. Functional connectivity between the ECN and the left and right cerebellum lobule VI in the aftermath of stress was higher in stressed participants (stress > no-stress left: *p*_fwe_ = 0.005, right, *p*_fwe_ = 0.037). There were no differences between controls and siblings. The contrast map is displayed at *p* < 0.001 uncorrected and overlaid onto a normalized anatomical scan for visualization purposes. For a color version, see this figure online. See Table 2 for cluster level inferences.

During the late aftermath of stress (RS2), we expected to find increased ECN functional connectivity in healthy controls. We found a significant increase in functional connectivity between the ECN and the left and right cerebellar lobule VI in all participants (stress > no-stress left *T* = 4.69; cluster size = 5656 mm^3^, *p*_whole−brain_ = 0.005, right *T* = 3.93, cluster size = 3392 mm^3^, *p*_whole−brain_ = 0.037) (Fig. 3). This effect did not differ between healthy controls and siblings (group × stress interaction *p* > 0.05). Table 2 lists the group results for these analyses.

**Table 2.** SN and ECN functional connectivity during acute stress (RS1) and the aftermath of stress (RS2)

Scan session	Brain region	Peak *T* value	Cluster size (mm^3^)	MNI coordinates (*x*,*y*,*z*)
SN				
RS1	*Group* *×* *stress interaction*			
	Insula, R	4.10	1120*††	40, −12, 8
	*Stress > no-stress*			
	*–*			
	*No-stress > stress*			
	*–*			
	*Con-stress > Con-no-stress*			
	Insula, R	3.88	896*†	34, −8, −12
	*Con-stress > Sib-stress*			
	Insula, R	3.43	24^#^	40, −2, 16
		3.30	32^#^	38, −8, 18
RS2	*Group* *×* *stress interaction*			
	Insula, L	3.82	400†	−38, −18, 6
		3.54	168^#^	−38, −2, −12
	*Stress > no-stress*			
	*–*			
	*No-stress > Stress*			
	Insula, L	3.97	232*†	−34, 8, 14
	*Sib-no-stress > Sib-stress*			
	Insula, L	3.85	288†	−34, 6, 12
		3.45	368†	−40, −18, 2
ECN				
RS1	*Group* *×* *stress interaction*			
	*–*			
	*Stress > no-stress*			
	*–*			
	*No-stress > stress*			
	–			
RS2	*Group* *×* *stress interaction*			
	*–*			
	*Stress > no stress*			
	Cerebellum lobule VI, L	4.69	5656**	−20, −56, −32
	Cerebellum lobule VI, R, extending to fusiform gyrus	3.93	3392*	32, −40, −20
	*No-stress > stress*			
	*–*			

MNI, Montreal Neurological Institute; R, right; L, left; Sib, sibling of schizophrenia patient; Con, control participant.

All effects are analyzed using cluster-level statistics, using a cluster-forming threshold of *p* < 0.001 uncorrected at the voxel level. For pairwise comparisons, only contrasts yielding significant clusters are displayed. ** *p* < 0.01 (whole brain corrected), * *p* < 0.05 (whole brain corrected); †† *p* < 0.01 (small-volume corrected for region of interest); † *p* < 0.05 (small-volume corrected for region of interest); ^#^ *p* < 0.1 (small volume corrected for region of interest).

Reported cluster sizes represented the size of a significant cluster after corrected for a small volume unless the cluster was significant at the whole brain level only.

Together, these results show no effect on ECN connectivity during acute stress, and a comparable increase in ECN-cerebellar connectivity in siblings and controls during the aftermath of stress.

## Discussion

This is the first study that compared the rapid and delayed effects of stress on resting-state functional connectivity in healthy controls and siblings of schizophrenia patients, a group that is at increased risk for a wide range of stress-related psychopathology. We aimed to (1) investigate the acute and delayed effects of stress on functional connectivity of the SN and the ECN as proposed by Hermans and colleagues (Hermans et al., 2014) and (2) investigate whether and when differences existed between controls and siblings. We found that stress causes a rapid increase in functional connectivity within the SN in healthy controls, while siblings of schizophrenia patients failed to upregulate SN functional connectivity in response to stress. Furthermore, we found reduced SN functional connectivity and increased connectivity between the ECN and the cerebellum in the aftermath of stress in both controls and siblings, with an additional decrease in SN-posterior insula connectivity in siblings. These findings demonstrate time-dependent opposing effects of stress on SN functional connectivity in healthy controls, and reveal for the first time that the neural dynamics in siblings of schizophrenia patients are primarily affected during the acute phase of stress.

### Differential effects of stress on salience network connectivity in siblings of schizophrenia patients

In line with the model proposed above, acute stress resulted in a rapid increase in functional connectivity between the right insula and the rest of the SN in healthy controls. Several studies have revealed immediate increases in the SN during or directly after stress (see Hermans *et al*. 2014; van Oort *et al*. 2017, for reviews). For example, immediately after stress, functional connectivity between the amygdala and other SN regions such as the insula and dACC was shown to be increased (van Marle et al., 2010). However, not all studies found increases in connectivity with the insula. A recent resting-state study found increased functional connectivity between the amygdala and the medial prefrontal cortex during resting state immediately after a stress induction paradigm, specifically in participants with strong cortisol responses (Quaedflieg et al., 2015). The insula is at the intersection of the cognitive, homeostatic, and affective systems, facilitating the bottom-up filtering of potential salient stimuli and, once a stimulus is detected, engaging other brain areas to initiate an appropriate response while disengaging areas that are not immediately relevant (Menon & Uddin, 2010). Therefore, our finding of increased SN-insula connectivity indicates an elevation in vigilance which may serve to quickly respond to changes in the environment.

In contrast to the SN effect in healthy controls, we found no increase in SN functional connectivity during acute stress in siblings of schizophrenia patients. There are at least two possible explanations for this finding. First, siblings of schizophrenia patients may be unable to upregulate SN functional connectivity in response to stress, while SN functional connectivity during baseline conditions is unaffected. Alternatively, SN functional connectivity may be chronically elevated in siblings and, as a consequence, a further increase in SN connectivity in stressful situations is compromised. The first explanation is supported by follow-up fMRI analyses between the four groups: SN connectivity during acute stress was lower in siblings compared to healthy controls, indicating impairments in salience processing and adequate response selection during stress. However, our finding of increased alpha-amylase levels in siblings during no-stress conditions appears more consistent with the second explanation. Alpha-amylase is an indirect marker of adrenergic activity (van Stegeren et al., 2006), and higher baseline concentrations, which did not further increase in response to stress, point towards higher basal sympathetic/noradrenergic tone. Supporting hypervigilance in mental illness, early work revealed an increased central noradrenergic output in schizophrenia patients that predict relapse (Maas et al., 1993; van Kammen et al., 1994; van Kammen & Kelley, 1991), and the effects of atypical antipsychotics on symptom reduction partly act through alpha-adrenergic receptors (Svensson, 2003). Possibly, a combination of the two explanations underlies the reduced responsiveness of the SN to stress in siblings. Elevated tonic norepinephrine levels in siblings may reduce detectable changes in phasic firing of noradrenergic neurons in the locus coeruleus (Aston-Jones & Cohen, 2005; Vazey, Moorman, & Aston-Jones, 2018), and lead to an attenuation of SN responses to stressors. We therefore hypothesize that a dynamic co-activation of the insula with the rest of the SN is necessary to respond adequately to acute stressors, and that in siblings, compromised SN upregulation due to an attenuated dynamic range in the sympathetic/noradrenergic response may constitute a vulnerability factor for the development of psychopathology.

In contrast to these acute effects, the late aftermath of stress is important for the normalization of emotional reactivity and higher order cognitive processes of the event. We found reduced functional connectivity between the right insula and the rest of the SN during the aftermath of stress in both controls and siblings, confirming the hypothesis of opposing effects of stress on SN connectivity during the acute and aftermath phases of stress (Hermans et al., 2014). The late phase did not differ between the two groups, indicating that network connectivity during the aftermath of stress is not affected in siblings. However, siblings showed an additional reduction in functional connectivity between a more posterior part of the insula and the rest of the SN during the late aftermath of stress. Interestingly, this area overlapped with the area that increased during the acute stress phase in healthy controls only. The anterior and posterior parts of the insula are in general both activated during salient stimuli processing, but they have different input and output connections and play different roles in saliency. Through connections between the anterior insula and the motor cortex, activating the SN initiates rapid response selection, while the integration of homeostatic sensory signals (such as thermoregulation) is regulated through connections with the mid and posterior insula (Craig, 2009; Menon, 2011). During the acute phase of stress, siblings did not show an increase in connectivity with the posterior insula, but apparently they do reduce this connection during the aftermath of stress, implicating a similar downregulation of this area after stress in controls and siblings and a lack of increased connectivity with this area during stress in siblings.

### Time-dependent effects of stress on executive control network functional connectivity

In contrast to the proposed model by Hermans et al. (2014), we found no reduced ECN functional connectivity during acute stress. A possible explanation is that the ECN might have been insufficiently activated to be affected by stress, given that we measured functional connectivity during rest, unlike previous studies that investigated ECN functional activity during cognitive tasks (Cousijn, Rijpkema, Qin, van Wingen, & Fernández, 2012; Oei, Tollenaar, Spinhoven, & Elzinga, 2009; Qin et al., 2009). Another explanation is that intrinsic communication of the ECN is not affected, but that the time-locking of activity to external events in accordance with task demands is altered during acute stress, as proposed by a recent study that found effects of aversive movie viewing on SN, but not ECN functional connectivity (Young et al., 2017).

Previous studies found that ECN functioning is increased in the late aftermath of stress, presumably through genomic actions of cortisol (Henckens, van Wingen, Joëls, & Fernández, 2011, 2012b; Hermans et al., 2014). Here, we found increased functional connectivity between the ECN and left and right lobule VI of the cerebellum, an area which co-activates with prefrontal and parietal regions during cognitive tasks such as language processing, working memory and executive function, even if a motor response is not required (Stoodley, 2012). Damage to the cerebellum further leads to deficits in working memory, indicating that cognitive processes partly depend on the engagement of cortico-cerebellar circuits. These results suggest that both healthy controls and siblings increase executive functioning during stress recovery, but it should be noted that we did not find an effect on core ECN regions and therefore the role of the cerebellum in stress must be further investigated in future studies. Concluding, we found that stress only affected ECN functional connectivity with the cerebellum in the aftermath of stress, with no differences between healthy controls and siblings of schizophrenia patients.

### Strengths and limitations

To the best of our knowledge, this is the first study to investigate the early and late effects of stress on SN and ECN functional connectivity in healthy controls and at-risk individuals. An important strength is that we used resting-state functional connectivity analyses which allow for repeated measurements without possible learning effects. Another strength of the study is that we induced stress using the TSST, a stress task that evokes the strongest cortisol response of all laboratory stressors (Skoluda et al., 2015). Reduced SN connectivity in the aftermath of stress is presumably driven by the genomic effects of cortisol, and therefore this finding might not have been detected if another stressor was used. On the other hand, moving the participants from the TSST room to the MRI scanner induces a delay, resulting in lower adrenergic activity during scanning. Therefore, stronger effects on network connectivity during acute stress might have been observed if we had chosen a different stressor, such as the presentation of aversive movie clips (Young et al., 2017), the cold pressor task (Blandini, Martignoni, Sances, Bono, & Nappi, 1995), or the montreal imaging stress task (Dedovic et al., 2005). Furthermore, we obtained the mean time course of each entire network, which was used as a regressor of interest in the first-level analyses. It is therefore possible that changes during and after stress are driven by one of the seed regions of the network. Moreover, it should be noted that we found effects in SN functional connectivity in opposing hemispheres during the acute and late phases of stress. However, since the conventional statistical parametric mapping approach does not allow for a direct comparison between the left and right hemispheres, this does not warrant conclusions about lateralization regarding the different stress phases. Furthermore, while the use of resting-state analyses has it benefits, it is difficult to make inferences about the effects of stress on responses to the environment. A previous study, however, found a strong overlap between task-related brain networks and networks during rest (Mennes et al., 2010), and interindividual differences during rest also correspond with interindividual differences during tasks (Smith et al., 2009). This implies that the observed responses to stress during rest might also be there during tasks. An important limitation is that our sample consisted of males only, and our results are therefore not generalizable to females. Given that there are gender-related differences in behavioral and neural responses to stress (Bangasser, Eck, & Ordoñes Sanchez, 2019; Kelly, Tyrka, Anderson, Price, & Carpenter, 2008; Lighthall et al., 2012), future studies should compare males to females directly.

## Conclusion

We aimed to investigate whether at-risk individuals differ from healthy controls in the temporal dynamics of networks involved in the central stress response. Our results show similarities during the aftermath of stress: both groups showed the expected changes in brain functioning during stress recovery. However, the groups differed during the acute phase of the stress response: at-risk individuals lacked the increase in neural resources necessary to quickly and adequately cope with a stressor, possibly as a consequence of chronically elevated catecholaminergic activity. Our results therefore offer a new explanation for the increased susceptibility for the development of stress-related psychopathology in this at-risk group.

## Supplementary material

For supplementary material accompanying this paper visit https://doi.org/10.1017/S0033291719004033. click here to view supplementary material
