# Supplementary material for: Disrupted upregulation of salience network connectivity during acute stress in siblings of schizophrenia patients
Source: Psychol Med. 2020 Jan 16;51(6):1038–48. doi: 10.1017/S0033291719004033 (PMC8161434; doi:10.1017/S0033291719004033)
Supplement: Supplementary file 1 [file S0033291719004033sup001.docx]

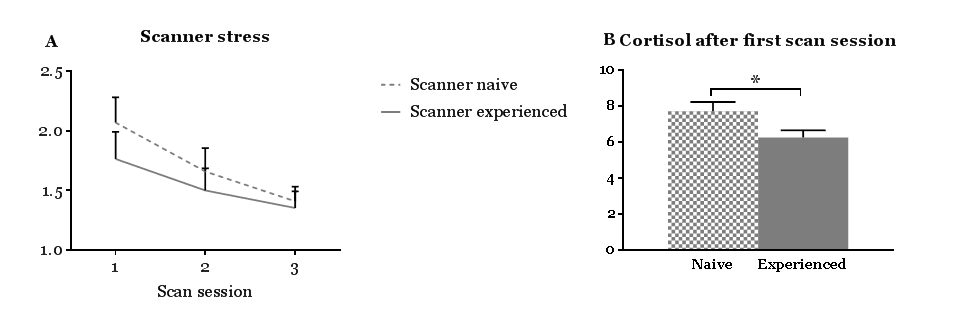


**Figure S1 | Subjective scanner stress before each scan session (A) and cortisol in nMol/L directly after the first scan session (B) across all participants.** Scanner naïve = never been in an MRI-scanner, scanner experienced = been in an MRI-scanner before.
